# Monocyte Subset Dynamics in Human Atherosclerosis Can Be Profiled with Magnetic Nano-Sensors

**DOI:** 10.1371/journal.pone.0005663

**Published:** 2009-05-22

**Authors:** Moritz Wildgruber, Hakho Lee, Aleksey Chudnovskiy, Tae-Jong Yoon, Martin Etzrodt, Mikael J. Pittet, Matthias Nahrendorf, Kevin Croce, Peter Libby, Ralph Weissleder, Filip K. Swirski

**Affiliations:** 1 Center for Systems Biology, Massachusetts General Hospital and Harvard Medical School, Boston, Massachusetts, United States of America; 2 Cardiovascular Division, Department of Medicine, Brigham & Women's Hospital, Boston, Massachusetts, United States of America; 3 Center for Excellence in Vascular Biology, Brigham & Women's Hospital, Boston, Massachusetts, United States of America; 4 Department of Systems Biology, Harvard Medical School, Boston, Massachusetts, United States of America; 5 Department of Radiology, Klinikum Rechts der Isar, Technische Universität München, Munich, Germany; Massachusetts General Hospital/Harvard University, United States of America

## Abstract

Monocytes are circulating macrophage and dendritic cell precursors that populate healthy and diseased tissue. In humans, monocytes consist of at least two subsets whose proportions in the blood fluctuate in response to coronary artery disease, sepsis, and viral infection. Animal studies have shown that specific shifts in the monocyte subset repertoire either exacerbate or attenuate disease, suggesting a role for monocyte subsets as biomarkers and therapeutic targets. Assays are therefore needed that can selectively and rapidly enumerate monocytes and their subsets. This study shows that two major human monocyte subsets express similar levels of the receptor for macrophage colony stimulating factor (MCSFR) but differ in their phagocytic capacity. We exploit these properties and custom-engineer magnetic nanoparticles for ex vivo sensing of monocytes and their subsets. We present a two-dimensional enumerative mathematical model that simultaneously reports number and proportion of monocyte subsets in a small volume of human blood. Using a recently described diagnostic magnetic resonance (DMR) chip with 1 µl sample size and high throughput capabilities, we then show that application of the model accurately quantifies subset fluctuations that occur in patients with atherosclerosis.

## Introduction

Circulating monocytes in humans fall into subsets typically identified by expression of the LPS receptor CD14 and the Fcγ receptor-III CD16. CD14^+^CD16^lo^ monocytes predominate in the blood and express high levels of the CCL2 (MCP-1) receptor CCR2 while CD14^lo^CD16^hi^ monocytes are less abundant and express higher levels of the fractalkine receptor CX_3_CR1. These expression patterns suggest differential tissue tropism, and indicate commitment of circulating monocytes for specific functional fates [1]–[4].

Atherosclerosis, a major cause of myocardial infarction, peripheral arterial disease, and stroke, is characterized by continuous accumulation of monocytes in the arterial intima [5], [6]. In the circulation, CD16^hi^ monocyte counts rise in patients with coronary artery disease [7], although the biological significance of this finding requires further investigation. Recent studies in animals have promoted the idea, however, that some monocyte subsets accentuate while others attenuate disease [8]. In this context, disease progression may be characterized by a subset imbalance that favors an inflammatory cell population. In hyperlipidemic atherosclerotic mice for example, one monocyte subset (Ly-6C^hi^; likely corresponds to CD16^lo^ in human [1]) preferentially accumulates in growing atheromata and differentiates into macrophages while another subset (Ly-6C^lo^; CD16^hi^ in human) accumulates less and likely differentiates to dendritic cells [9], [10]. The possibility that monocytes participate divergently in lesion growth necessitates evaluation of how findings obtained in animals translate to humans, and whether monocyte subsets represent therapeutic targets and prognostic biomarkers.

High-throughput profiling of peripheral monocytes in humans requires tools of exceptional selectivity and sensitivity. Clinically, automated blood differentials quantify circulating monocytes but do not inform on subsets [11], [12], and no other diagnostic tools are routinely available that can delineate how specific subsets fluctuate in population samples. In this study, we profiled phenotypic and functional characteristics of human monocyte subsets and focused on two distinguishing features: shared expression of MCSFR among subsets and differential capacity of monocyte subsets for phagocytosis. We custom-engineered novel magnetic nanoparticles and, using a recently developed diagnostic magnetic resonance (DMR) chip technology [13], simultaneously profiled monocyte and monocyte-subset changes for use in patients with atherosclerosis.

## Results

### Divergent phenotypic and functional properties of human monocyte subsets furnish prospective labeling targets

**Figure 1:**
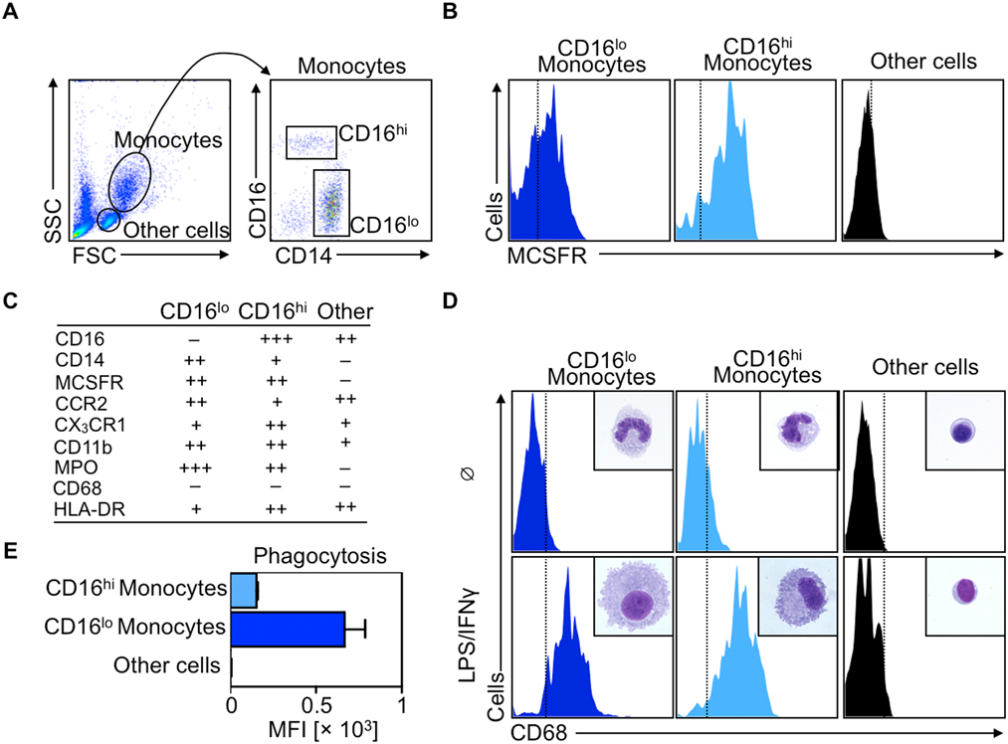
Human monocyte subsets differ phenotypically and functionally. A. Flow cytometry dot plots show forward scatter (FSC) versus side scatter (SSC) of mononuclear cells obtained from fresh blood. A monocyte gate is drawn and monocyte subsets are identified according to their CD14 and CD16 expression profile. B. Histograms depict MCSFR expression of CD16^lo^ monocytes, CD16^hi^ monocytes and other cells (mostly lymphocytes). C. Table summarizes relative expression profiles of selected markers for CD16^lo^, CD16^hi^ monocytes and other cells. D. Representative histograms and H&E cytospin preparations show CD68 expression and morphology of CD16^lo^, CD16^hi^ monocytes and other cells freshly isolated (□) or after in vitro culture for 6 days with LPS/IFNγ. E. Bar graph depicts ex vivo phagocytosis of fluorescently labeled latex beads in CD16^lo^, CD16^hi^ monocytes and other cells (n = 4).

Monocyte heterogeneity may represent an as yet unexplored target for imaging and treatment [8], [14], but currently no clinical assays can simultaneously discriminate between monocyte subsets. Thus, we first focused on the biology of human monocyte subsets with the aim of uncovering a phenotype or function readily exploitable for specific and selective targeting. Mononuclear leukocytes were obtained from peripheral blood of healthy volunteers. The procedure uses density-gradient centrifugation, and thus enriches for mononuclear cells by removing neutrophils and other granulocytes. Monocytes are 10–30 µm in diameter, are therefore larger than lymphocytes, and occupy a distinct gate on a forward scatter/side scatter (FSC/SSC) flow cytometric dot plot (Fig. 1A). Labeling with antibodies against CD14 and CD16 allows identification of two monocyte subsets: a dominant CD14^+^CD16^lo^ (thereafter referred to as CD16^lo^) population and a minor CD14^lo^CD16^hi^ (CD16^hi^) population (Fig. 1A), as previously reported [15], [16]. Both subsets bear MCSFR (also known as CD115) (Fig. 1B) but fall into two subsets identified by distinct expression profiles of trafficking (CCR2, CX_3_CR1) and myeloid function/differentiation (CD11b, MPO, CD68, HLA-DR) markers (Fig. 1C). Importantly, neutrophils, which were absent in the preparations, also express markers such as CD14, CD16, CD11b and MPO, but not MCSFR. When cultured for 6 days with LPS and IFNγ, mediators that promote the acquisition of the M1-macrophage phenotype, both subsets, but not other cells, acquire morphologic characteristics of mature macrophages, and both subsets display increased levels of the macrophage marker CD68 (Fig. 1D). Similar macrophage morphology and expression of CD68 occur in cells cultured with the M2-phenotype-promoting mediators IL-4/IL-13 (data not shown). However, freshly-isolated monocyte subsets phagocytose fluorescently-labeled latex beads differently: both subsets are positive for bead uptake, but the cellular bead concentration, as assessed by the beads' mean fluorescent intensity (MFI), is significantly higher in the CD16^lo^ population, indicating higher phagocytosis by this subset compared to its CD16^hi^ counterpart (Fig. 1E). Altogether, these data identify at least two promising targets to label and track human monocytes and their subsets. MCSFR expression is a potential candidate to selectively target monocytes because monocytes express MCSFR uniquely and at similar intensities. Phagocytosis lends itself to discrimination between monocyte subsets because targeting phagocytosis is simpler than targeting differential expression of membrane-bound proteins.

### Targeted fluorescent iron-oxide nanoparticles allow discrimination of monocyte subsets optically

**Figure 2:**
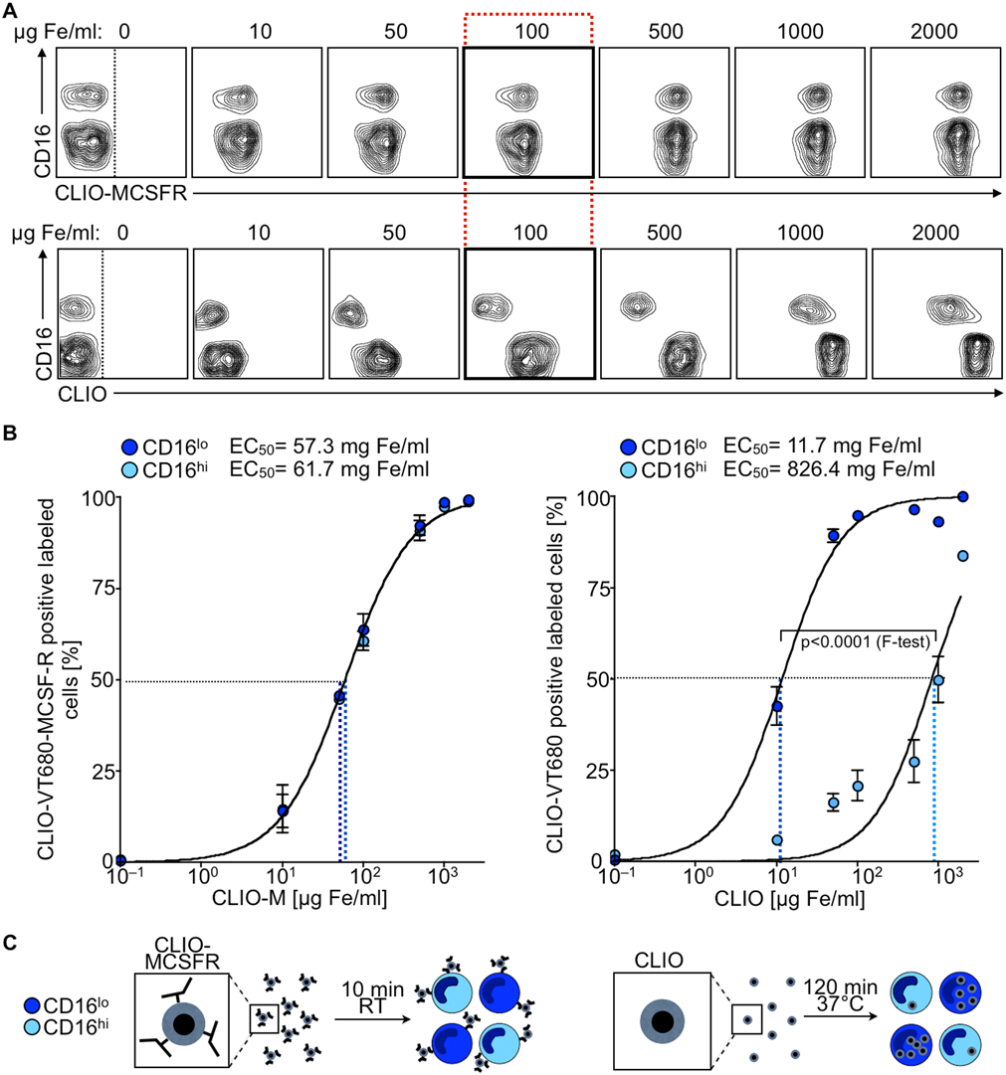
Nano-sensors discriminate between monocyte subsets optically. A. Representative flow cytometry contour plots of human monocytes labeled with increasing concentrations of fluorescent superparamagnetic nano-particles CLIO-MCSFR (top row) and CLIO (bottom row). To discriminate between subsets the mean fluorescent intensity of the particle (x-axis) is plotted against expression of CD16 (y-axis). B. Fe concentrations for both nano-particles (CLIO-MCSFR and CLIO) are log-transformed (x-axes) and plotted against % of positive labeled cells (y-axes). A sigmoidal dose-response curve is generated to calculate the corresponding EC_50_ (nano-particle concentration at which 50% of each monocyte subset is labeled). N = 4. C. Principle of the assay. The principle postulates that equal binding of subsets with CLIO-MCSFR will occur after 10 min at RT while incubation of subsets with CLIO at 120 min at 37°C will result in preferential uptake of the particle by CD16^lo^ monocytes.

Having identified suitable targets to phenotype human monocytes, we next considered whether functionalized nanoparticles report on these targets with sufficient sensitivity and selectivity. We engineered a putative monocyte-targeted nanoparticle by covalently attaching antibodies against MCSFR to cross-linked iron oxide (CLIO) which is a dextran-coated, superparamagnetic nanoparticle [17]. The advantages of a nanoparticle-based strategy compared to antibody alone include the ability to conduct magnetic resonance sensing in optically turbid media such as blood, and the likely improvement in the stoichiometry of MCSFR targeting because, on average, a single CLIO molecule can bear 2–3 MCSFR antibodies. Further covalent attachment of the NIR fluorochrome VT680 allows for an independent, optical read-out. Incubation of mononuclear cells for 10 min at room temperature (RT) with increasing doses of fluorescently-tagged CLIO-MCSFR leads to similar labeling of both CD16^hi^ and CD16^lo^ monocyte subsets across all concentrations (Fig. 2A), with no detectable labeling of other cells (data not shown) and no toxicity at doses up to 1000 µg Fe/ml as detected by Trypan blue and Annexin V staining, in accord with the literature [18], [19]. The labeling is antibody-specific because incubation of non-derivatized fluorescent CLIO for the same duration and temperature does not result in significant particle uptake (data not shown). The high MFI, especially at doses of 500–2000 µg Fe/ml suggests enhanced binding by the MCSFR antibody. Notably, the dose of 100 µg Fe/ml approximates Fe concentrations in human plasma detected shortly after intravenous injection of clinically approved doses of nanoparticles such as MION [20].

To test for phagocytosis, we hypothesized that non-derivatized CLIO, when incubated with cells at physiological conditions for a longer period of time, will report on differences in phagocytic capacity between subsets. In contrast to the similar labeling achieved with fluorescent CLIO-MCSFR, incubation of mononuclear cells for 120 min at 37°C with increasing doses of fluorescent CLIO leads to preferential uptake of the agent by CD16^lo^ monocytes at all concentrations tested (Fig. 2A), reflecting the heightened capacity of these cells for phagocytosis. Leukocytes other than monocytes do not accumulate the particle significantly (data not shown).

Calculation of the concentration of the nanoparticles at which 50% of the cells are labeled (EC_50_) used an equation derived from fitting of a sigmoidal concentration-dependence relationship (Fig. 2B). CLIO-MCSFR yielded a similar calculated EC_50_ between subsets: 57.3 mg Fe/ml for CD16^lo^ and 61.6 mg/Fe/ml for CD16^hi^ monocytes (p = 0.3845). In contrast, unconjugated CLIO yielded a significantly different calculated EC_50_ between the subsets: 11.7 mg Fe/ml for CD16^lo^ and 826.4 mg Fe/ml for CD16^hi^ subsets (p<0.0001). Thus, the equal labeling of monocytes with fluorescent CLIO-MCSFR permits identification of monocytes while differential uptake of fluorescent CLIO effectively discriminates between monocyte subsets (Fig. 2C).

### Monocyte subset fluctuations can be resolved with a diagnostic magnetic resonance (DMR)-chip and modeled mathematically for enumeration studies

**Figure 3:**
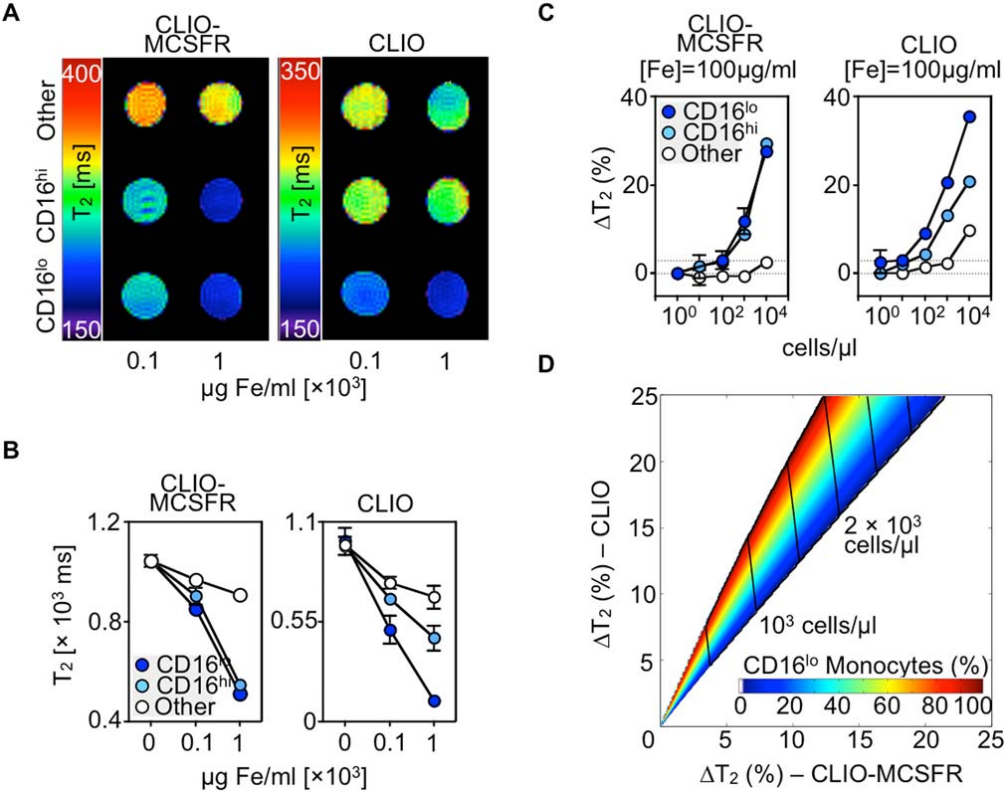
Ex-vivo Nuclear Magnetic Resonance generates an enumerative mathematical model for monocyte subsets. A. Representative NIH-color coded map generated from T_2_-weighted Magnetic Resonance Imaging. Data show equal number of CD16^lo^, CD16^hi^ monocytes and other leukocytes labeled with two CLIO-MCSFR (left panel) and CLIO (right panel) concentrations. B. T_2_ measurements detected with a conventional benchtop-relaxometer. Data show equal number of CD16^lo^, CD16^hi^ monocytes and other leukocytes labeled with two CLIO-MCSFR (left panel) and CLIO (right panel) concentrations. N = 3–5. Mean±SEM. C. T_2_ changes detected with a diagnostic magnetic resonance (DMR) chip. Data show increasing number of CD16^lo^, CD16^hi^ monocytes and other leukocytes labeled with one CLIO-MCSFR (left panel) and CLIO (right panel) concentration. N = 3. Mean±SEM. D. Two-dimensional T_2_ map derived from data in C to simultaneously enumerate total monocyte numbers and subset proportions. Model combines T_2_ changes for CLIO-MCSFR (x-axis) and CLIO (y-axis). Changes in predicted monocyte number are demarcated with vertical lines while the rainbow region defines monocyte subset fluctuations.

An emerging application of nanotechnology for clinical high-throughput screening and diagnosis utilizes a chip-based diagnostic magnetic resonance (DMR) system [13]. The sensitivity of DMR technology permits analysis of rare targets in sample volumes ≤1 µl with few or no sample purification. Before testing with the DMR chip, we first sought to determine with conventional approaches the feasibility of discriminating between subsets by magnetic resonance. Equal numbers of sorted monocyte subsets were labeled at different Fe concentrations with either CLIO-MCSFR or CLIO and were investigated by Magnetic Resonance Imaging (MRI) with a multi-slice multi-echo sequence at 7 T (Fig. 3A) and with a conventional bench top relaxometer at 0.5 T (Fig. 3B). Leukocytes other than monocytes were used as controls. Both CD16^lo^ and CD16^hi^ monocyte subsets experienced a similar decrease in spin-spin relaxation time (T_2_) when labeled with CLIO-MCSFR. The decrease was concentration-dependent, significantly less pronounced than in other cells, and evident with both methods. In contrast, CD16^lo^ monocytes showed an accentuated decrease in T_2_ values compared to CD16^hi^ monocytes when labeled with CLIO alone. The T_2_ decrease for CD16^hi^ monocytes was intermediate when compared to CD16^lo^ and control leukocytes, and was likewise concentration-dependent and method-independent. These data indicate the feasibility of discriminating between subsets with targeted superparamagnetic nanoparticles, and suggest that the DMR chip, which is expectedly more sensitive and requires smaller samples volumes, provides a promising approach.

To test the DMR chip for monocyte sensing we next incubated a priori known numbers of monocyte subsets with 100 µg Fe/ml of either CLIO-MCSFR or CLIO and measured T_2_ changes (ΔT_2_) at different cell concentrations. The DMR chip detects as few as 100 monocytes (10^5^ cells/ml) resuspended in human serum, with robust read-outs at higher monocyte concentrations (Fig. 3C). This sensitivity suffices to detect monocytes in healthy controls in volumes of blood as low as 1 µl. Both CD16^hi^ and CD16^lo^ monocyte subsets show similar ΔT_2_ when incubated with CLIO-MCSFR. However, ΔT_2_ are higher in CD16^lo^ monocytes when the phagocytic capacity is probed with CLIO. Labeling of leukocytes other than monocytes is minimal with either particle.

On the basis of these data, we formulated an enumerative mathematical model for monocyte populations and their subset proportions. First, we determined the cellular relaxivity (*R_ij_*) for each cell type (*i*: CD16^hi^, CD16^lo^ and others) and nanoparticle (*j*: CLIO-MCSFR and CLIO) combination by fitting the titration curve (Fig. 3C) into Δ(1/T_2_)*_j_* = *R_ij_*·*N_i_*, where *N_i_* is the concentration of a given cell type (*i*). When a sample with heterogeneous cell composition is probed with a nanoparticle (*j*), the total Δ(1/T_2_)*_j_* is approximated as the sum of the individual contributions by each cell type (*i*): Δ(1/T_2_)*_j_* = ∑ *R_ij_*·*N_i_*. We thus obtained two equations of Δ(1/T_2_)*_j_* for each nanoparticle (*j*: CLIO-MCSFR or CLIO) which can be solved to determine *N*_i_ for each monocytes subset. Note that the contribution from other cells can be exactly compensated provided that *N*_others_ is known. Otherwise, we could use the highest Δ(1/T_2_) for other cells (Fig. 3C) to obtain a conservative estimation on monocyte populations. Applying the method, we could then construct a 2-dimensional ΔT_2_ map (Fig. 3D) that can be used to simultaneously determine the total monocyte population and the subset proportions from observed T_2_ changes.

### Patients with atherosclerosis have an altered monocyte subset profile detectable by a two-dimensional DMR-Chip-based assay

**Figure 4:**
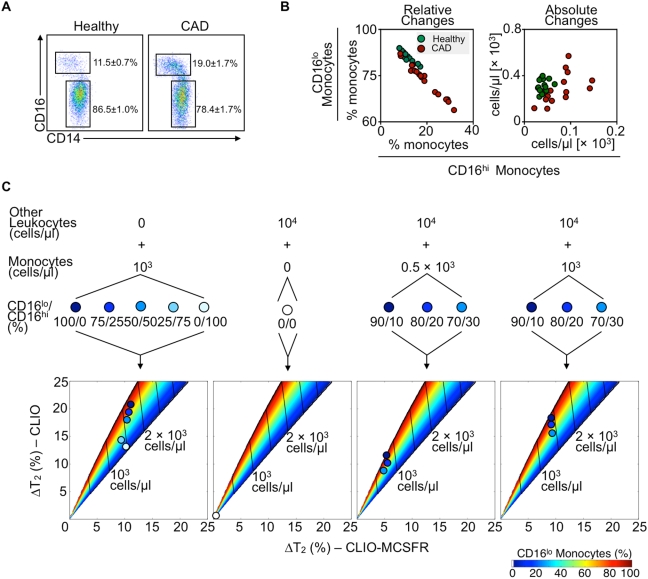
Magnetic nano-sensors enumerate monocyte subset variations that occur in atherosclerotic patients. A. Representative flow cytometry dot plots of monocyte subsets from healthy volunteers and patients with documented coronary artery disease (CAD). Numbers depict percentage of subsets in both groups. Mean±SEM. B. Plots depict percentage (left plot) and absolute numbers (right plot) of CD16^lo^ and CD16^hi^ monocytes from healthy volunteers (green dots) and patients with CAD (red dots). C. Validation of the enumerative mathematical model with varying number and percentage of monocyte subsets. Data show goodness of fit of defined numbers of monocytes alone (left panel), other leukocytes (middle-left panel) and combinations of leukocytes and monocytes (right two panels). Different proportions of monocyte subsets (CD16^lo^/CD16^hi^ (%)) are color-coded and their fit is depicted on the two-dimensional T_2_ maps.

A high-throughput assay that enumerates cells and discriminates between subsets requires that the cells in question fluctuate within the assay's dynamic detection range. As a proof of principle, we investigated monocyte numbers and proportions in two cohorts: healthy volunteers and patients with coronary artery atherosclerotic disease (CAD) undergoing cardiac catheterization at Brigham and Women's Hospital. Blood from 12 healthy volunteers contains, on average, 86.5±1.0% of CD16^lo^ and 11.5±0.7% CD16^hi^ monocytes, whereas blood from 18 patients with CAD contains 78.4±1.7 CD16^lo^ and 19.7±1.7% CD16^hi^ monocytes (Fig. 4A). Comparison of the relative and absolute changes between healthy volunteers and patients with CAD reveals a greater range in patients with CAD both in terms of proportion and monocyte cell number (Fig. 4B). Elevation of CD16^hi^ monocytes is significant proportionally (p = 0.0016) and in absolute numbers (p = 0.0192). While larger and better-controlled clinical studies are needed to determine this finding's significance – for example, whether altered monocyte subset numbers portend prognosis in patients with atherosclerosis – this preliminary enumeration reveals a sufficient sensitivity of a DMR-chip assay to detect values observed in the clinic.

We next evaluated the accuracy of the DMR assay to profile monocyte populations in complex and physiological cell compositions. This is important, as it would reveal whether the method has clinical, high throughput potential. Samples were incubated with CLIO-MCSFR or CLIO, and observed T_2_ changes were plotted on the two dimensional ΔT_2_ map already formulated and shown in Fig. 3D. First, we tested the accuracy of the model to quantify purified monocytes at various subset proportions (Fig. 4C, left panel). T_2_ changes show strong agreement with the model over the proportional range (%CD16^lo^/%CD16^hi^: 100/0, 75/25, 50/50, 25/75, 0/100). As a negative control, leukocytes other than monocytes were incubated and, as expected, the observed ΔT_2_ values are negligible (Fig. 4C, center left panel). However, monocytes are outnumbered by other leukocytes in blood and subsets fluctuate across a range of ∼30% (Fig. 4B). We therefore evaluated samples in which physiological monocyte concentrations (0. 5×10^3^ and 10^3^ cells/µl) at various physiological subset proportions (%CD16^lo^/%CD16^hi^: 90/10, 80/20, 70/30) were mixed with other leukocytes, also at physiological concentrations (10^4^ cells/µl). T_2_ changes for CLIO-MCSFR fit into the model's prediction, although the model slightly overestimates total monocyte counts, possibly due to the increased sample viscosity and the attendant T_2_ shortening. For a given cell population, the T_2_ changes for CLIO correctly reports the subset ratios. These experiments demonstrate that the DMR assay enumerates monocyte subsets in human samples and measures ≤10% changes of monocyte subsets even when the monocytes represent only a rare leukocyte population. Importantly, the method detects cells in volumes much smaller than is required for flow cytometry.

## Discussion

This study reports the development and evaluation of an assay that enumerates circulating monocyte subsets in small sample volumes (1 µl). The assay detects small changes of rare leukocyte subset populations with sensitivities not possible with conventional clinical tools. The simultaneous enumeration of total monocytes and their relative proportion is feasible because of magnetic nanoparticles that target different cellular features and properties. The method described here is therefore customizable to profile various cellular phenotypes for comprehensive disease screening.

The recognition of the involvement of inflammation in atherosclerosis promises to lead to improved detection and treatment modalities for patients at risk for atherothrombotic events. This study focused on analysis of monocyte subset fluctuations that occur in patients with atherosclerosis as these cells participate critically in disease progression and can be obtained simply with blood withdrawals [3], [21]. Moreover, the observation that monocyte subsets differentially participate in experimental atherogenesis [9], [10], [22] underscores the need to assess the consequence of their fluctuations in humans. Some clues are already available: human coronary artery lesions contain macrophage subpopulations with different gene expression patterns [23], while CD16^lo^ monocytes accumulate lipids preferentially in vitro [24]. It remains unknown whether functional monocyte heterogeneity in atheromata arises from divergent stimuli encountered by infiltrating but uncommitted macrophage precursors, or whether functionally committed monocyte subsets accumulate in specific lesional niches, or at defined moments of plaque evolution, to influence plaque growth or stability. An altered monocyte subset profile in patients with atherosclerosis argues for the latter possibility, even if it does not preclude the former, and suggests that monocyte subsets may serve as biomarkers and targets for discriminate monocyte-based therapeutic intervention.

Future large scale studies that determine if and how blood monocyte profiles predict complications of atherosclerosis will benefit from sensitive and efficient diagnostic tools such as the nanoparticle-based DMR chip assay described here, particularly given the assay's relatively small sample needs, low cost, and high-throughput capability. Because the assay depends primarily on R2 relaxivities of the magnetic nanoparticles and the volume of the sample [13], [25], future efforts will include *de novo* synthesis of magnetic nanoparticles with higher R2 compared to conventional particles, and further miniaturization and improvement of the NMR-microfluidic chips. The next generation of DMR systems may detect single cells in a small volume of turbid and diverse media. Application of this technology may further identify that numerous pathologies other than atherosclerosis, such as cancer, HIV infection, sepsis, or kidney failure, carry a specific “monocyte-subset signature” [11], [26]–[32], and it will remain to be determined how such observations associate with disease prognosis and severity. Finally, the method should have even more widespread utility for detection and profiling of other rare cell populations.

## Materials and Methods

### Isolation of human monocytes

#### Ethics Statement

The protocol was approved by the Institutional Review Board at Brigham & Women's Hospital, Boston. Whole blood was obtained from healthy volunteers. CAD samples were obtained from patients undergoing cardiac catheterization at Brigham & Women's Hospital. Patients included in the protocol were diagnosed with coronary artery disease (CAD), defined by >70% stenosis in 1 or more epicardial coronary artery determined by coronary angiography. All donors gave written and informed consent. Fresh whole blood was drawn into heparinized collection tubes. To obtain leukocyte suspensions, whole blood was diluted 1∶1 with DPBS and 20 ml diluted blood was overlaid on a 15 ml density gradient (Ficoll-Paque Plus, density 1.077 g/ml, GE Healthcare, NJ) and centrifuged (20 min, 1600 rpm, 18°C). The mononuclear cell interphase was carefully isolated and washed 3 times with DPBS. Resuspended cell suspensions were counted using Trypan blue (Cellgro, Mediatech Inc., Manassas, VA).

### Cells

Cell suspensions were stained with the following antibodies (all from BD Bioscience, unless otherwise stated) at a final concentration of 1∶100: CD11b-APC-Cy7/ICRF44, CD14-PE/M5E2, CD16 PE-Cy7/3G8, CCR2-Alexa-647/48607, CX_3_CR1-FITC/2A91 (MBL International, Woburn, MA), MPO-FITC/2C7 (AbD Serotec, Raleigh, NC), HLA-DR-APC/L243, CD68-PE/Y1-82A, MCSFR-FITC/61708 (R&D Systems, Minneapolis, MN). Intracellular MPO staining was performed after fixation and permeabilization (BD Cytofix/Cytoperm, BD Bioscience, San Jose, CA). Cell phenotyping was performed using a LSRII Flow Cytometer (BD Bioscience, San Jose, CA) after appropriate compensations. For cell sorting, cells were labeled with CD14/CD16 and flow-sorted with a FACSAria (BD Bioscience, San Jose, CA). Purity of each monocyte subset population was >95% as determined by post-FACS flow-cytometric assessment. Non-monocyte control cells were sorted according to their Forward- and Side-Scatter profiles. These cells were ∼90% lymphocytes on post-FACS analysis and were therefore considered “other cells”; they do not include neutrophils or granulocyte that were depleted with density gradient centrifugation. For in-vitro differentiation into macrophages, FACS sorted monocyte subsets were cultured in 200 µl cultures for 6 days in 1640 RPMI (containing glutamine, supplemented with 10% heat-inactivated FCS, 1% Penicillin/Streptomycin, Cellegro, Washington, DC) with additional cytokine stimulation: with LPS at 100ng/ml (Sigma, Saint Louis, MO) and IFNγ at 1000 U/ml as well as with IL-4 and IL-13, both at 20 ng/ml (from R&D Systems, Minneapolis, MN). Cytokine-enriched medium was changed every two days. Viability of cell culture was assessed by trypan blue. Flow cytometric data were analyzed using FlowJo v.8.5.2 (Tree Star, Inc., Ashland, OR). For morphologic characterizations, sorted cells were prepared on slides by cytocentrifugation (Shandon, Inc., Pittsburgh, PA) at 10×g for 5 min, and stained with HEMA-3 (Fischer Scientific, Pittsburgh, PA).

### Phagocytosis assay

For the determination of differences in phagocytosis between both monocyte subsets, yellow-green labeled latex beads were used (Bead size 2.0 µm, Sigma, Saint Louis, MO). FACS-sorted monocyte subsets where incubated at a cell/bead ratio of 1/10 for 4 h at 37°C in RPMI 1640, supplemented with 1% Penicillin/Streptomycin and 10% heat-inactivated fetal calf serum (FCS). After incubation, free beads were washed from the cell suspension 3 times and cells were analyzed by flow cytometry.

### Magnetic nanoparticles

For optical assessment a Near-infrared fluorescent (NIRF) fluorochrome (VT680, Excitation 670±5 nm, Emission 688±5 nm, Visen Medical, Woburn, MA) was coupled to cross-linked iron oxide nanoparicles (CLIO) as previously described [33]. Fluorescent CLIO had the following properties: Size ∼ 30 nm, R1 = 28.8 s^−1^ mM^−1^ [Fe], R2 = 74.3 s^−1^ mM^−1^ [Fe]. For further experiments monoclonal MCSFR antibody (Clone 61708, R&D Systems, Minneapolis, MN) was coupled covalently to fluorescent CLIO. An average of 2.5 antibodies were immobilized per nanoparticle as determined with bicinchoninic acid assay. The resulting CLIO-MCSFR particles were protected from light and stored at 4°C. For uptake experiments non-derivatized CLIO was used (Center for Molecular Imaging Research, Massachusetts General Hospital, Charlestown, MA). For the detection of VT680, an LSR II Flow Cytometer was equipped with a 685/LP and 695/40 BP filter.

### Exposure of cell suspensions to magnetic nanoparticles

FACS sorted monocytes or unsorted leukocytes were plated at 100 K/200 µl in a 96 well plate. Cell labeling with iron-oxide nanoparticles was performed at different concentrations from 0 to 2000 µg Fe/ml in 1640 RPMI. Cell suspensions were incubated at the following conditions: CLIO-MCSFR (10 minutes, RT), CLIO (2 h, 37°C, humidified CO_2_ atmosphere). After the incubation period, cell suspensions were washed 3 times to separate labeled cells from unbound particles. For optical assessment by flow cytometry, cells were additionally stained with CD14 and CD16. After labeling, cells were counted again with trypan blue.

### Magnetic resonance sensing

FACS-sorted monocyte subsets and control cells were incubated with CLIO-MCSFR and CLIO at 100 µg Fe/ml. 10^5^ labeled cells were resuspended in 300 µl sucrose gradient solution (Ficoll-Paque Plus, density 1.077 g/ml, GE Healthcare, NJ) to prevent sedimentation of cells during sensing [34]. The Ficoll-cell suspension was subsequently embedded in an agarose-gel phantom, which minimizes susceptibility artifacts caused by interfaces with air or plastic. MR imaging was performed using a 7-T horizontal-bore scanner (Pharmascan, Bruker, Billerica, MA) and a volume coil in birdcage design (Rapid Biomedical, Wuerzburg, Germany). A T_2_ weighted multi-slice multi-echo sequence was used with the following parameters: TE = 8.8 ms, TR = 2330 ms, flip angle = 90 degrees excitation and 180 degrees refocusing, slice thickness = 1 mm, matrix 128×128, FOV 4×4 cm. NIH-color coded T_2_ maps were calculated using OsiriX (Geneva, Switzerland).

### Benchtop NMR relaxometer

For T_2_ measurements with a conventional Benchtop Relaxometer (Minispec mq20, Bruker BioSpin, Billerica, MA), FACS-sorted monocyte subsets and control cells were incubated with CLIO-MCSFR and CLIO at 100 µg Fe/ml. 100K of labeled cells were resuspended in 300 µl 1% Triton ×100 solution and T_2_ assessment was performed at 0.47 T (20 MHz).

### DMR assay

For rapid detection of monocyte heterogeneity in small sample volumes, we custom-designed a Diagnostic Magnetic Resonance (DMR) chip, as previously reported [13]. The DMR system consists of a solenoidal microcoil for NMR detection, a microfluidic channel for sample handling, a single-board NMR electronics and a small permanent magnet (0.5 T). The microcoil was embedded along with the microfluidic channel to achieve high filling factor (≈1) and thereby larger NMR signal. For DMR assay, FACS sorted monocyte subsets were incubated with CLIO-MCSFR and CLIO (100 µg Fe/ml) as described above. Labeled cells were resuspended in human serum and DMR measurements were performed with 1 µl sample volumes. T_2_ were measured using Carr-Purcell-Meiboom-Gill pulse sequences with the following parameters: TE = 4 ms, TR = 6 s; the number of 180° pulses per scan, 500; the number of scans, 8. For ΔT_2_ calculation, T_2_ differences were calculated between magnetically labeled and cell-number matched control samples. All measurements were performed in triplicate.

### Statistics

Data are expresses as Mean±SEM. For group comparisons Student's t test was used. For multiple comparisons ANOVA was used. For assessment of the EC_50_ a sigmoidal dose-response equation was chosen [Y = Bottom+(Top-Bottom)/1+10^∧^((Log EC_50_-X)*Hill Slope))], for comparison of the EC_50_ of the two different monocyte subsets F-test was used. When ΔT_2_ values are presented the T_2_ relaxation time of a sample with non-labeled cells, resuspended in human serum, is used as reference. Each measurement was carried out at least in triplicates, unless stated otherwise. P<0.05 was considered statistically significant. Statistical Analysis was performed with GraphPad Prism 4.0c for Mac (GraphPad Software, Inc, San Diego, CA).
